# Ropivacaine in oblique subcostal transversus abdominis plane block for laparoscopic sleeve gastrectomy: a randomized, double-blind clinical trial

**DOI:** 10.1590/acb415826

**Published:** 2026-11-09

**Authors:** Luís Eduardo Veras Pinto, Roclides Castro de Lima, João Nogueira, Almir Vieira Dibai, Caio Marcio Barros de Oliveira, Ed Carlos Rey Moura, Christian Lamar Scheibe, José Aparecido Valadão, Rioko Kimiko Sakata, Plínio da Cunha Leal

**Affiliations:** 1 Universidade Federal de São Paulo – Postgraduate Program in Interdisciplinary Surgical Science – São Paulo (SP), Brazil.; 2 Universidade Federal do Maranhão – Department of Medicine – São Luís (MA), Brazil.; 3 Universidade Federal do Maranhão – Postgraduate Program in Physical Education – São Luís (MA), Brazil.; 4 Hospital São Domingos – Department of Surgery – São Luís (MA), Brazil.

**Keywords:** Nerve Block, Gastrectomy, Postoperative Pain, Ropivacaine, Analgesia

## Abstract

**Purpose:**

To evaluate the analgesic efficacy, opioid consumption, and inflammatory response of oblique subcostal transversus abdominis plane (OSTAP) block with 0.2% ropivacaine versus control in patients undergoing laparoscopic sleeve gastrectomy (LSG).

**Methods:**

This is a prospective, randomized, double-blind, clinical trial. After standardized anesthetic induction, patients received a bilateral OSTAP block with 0.2% ropivacaine or 0.9% saline. Pain, rescue morphine, postoperative nausea and vomiting (PONV), Quality of Recovery-15 (QoR-15), enhanced recovery after bariatric surgery (ERABS) discharge criteria, and inflammatory markers were assessed.

**Results:**

Rest pain was similar between groups at all time points. During movement, a lower score was observed at 24 h in the ropivacaine group (RG), median 1 (0–6) *versus* 2 (0–6), but the difference was not statistically significant after adjustment (*p* = 0.024; Benjamini-Hochberg [BH] adjusted *p* = 0.096). Total morphine consumption was similar, 10 (2–50) *versus* 11 mg (1–30), *p* = 0.926, as were PONV, QoR-15 118 points, *p* = 0.717, diet resumption 98, ambulation 76, and ERABS discharge criteria. Interleukin-8 at T3 was numerically lower in the RG, 7.2 (0.6–27.1) *versus* 9.8 (1.3–18.6) pg/mL, without statistical significance after adjustment (*p* = 0.073; BH adjusted *p* = 0.142).

**Conclusion:**

OSTAP block with 0.2% ropivacaine was not associated with consistent differences in analgesic, recovery, or inflammatory outcomes.

## Introduction

Obesity is one of the most serious public health problems nowadays. More than one billion people worldwide live with obesity^1^. The World Obesity Federation estimates that the global prevalence of obesity more than doubled between 1990 and 2022, and projections indicate that the number of adults with obesity may reach 1.53 billion by 2035^2^. In Brazil, data from Telephone-based Surveillance System for Risk and Protective Factors for Chronic Diseases (Vigitel) show an increase in obesity prevalence from 11.8 to 25.7% between 2006 and 2024^3^.

Bariatric surgery has been established as an effective and durable intervention for the treatment of severe obesity and its associated comorbidities^4,5^. Among the available procedures, laparoscopic sleeve gastrectomy (LSG) consists of resection of approximately 80% of the greater curvature of the stomach, promoting loss of 50–70% of excess weight within up to five years, in addition to favorable hormonal changes such as reduced ghrelin levels and early GLP-1 and PYY signaling^6,7^. LSG can cause moderate to severe postoperative pain due to the use of multiple trocars, abdominal manipulation in individuals with a high body mass index, and elevated pneumoperitoneum pressure^8^.

Inadequately controlled postoperative pain prolongs recovery, increases length of hospital stay, raises the risk of thromboembolic and respiratory complications, and negatively affects patients’ quality of life^9^.

Furthermore, a recent systematic review reported that patients undergoing bariatric surgery are at risk of chronic opioid use^10^. Although opioids are potent analgesics, they are associated with significant adverse effects in the obese population, including respiratory depression, particularly in patients with obstructive sleep apnea, as well as nausea, vomiting, constipation, pruritus, sedation, and risk of dependence^11,12^.

The subcostal approach to the transversus abdominis plane (TAP) block, known as the oblique subcostal transversus abdominis plane (OSTAP) block, is particularly suitable for upper abdominal surgery, as it provides analgesic coverage of dermatomes T6-T10, including trocar insertion sites used in LSG^13-15^.

Ropivacaine is a particularly suitable option for infiltrative techniques in patients undergoing bariatric surgery, as it is an amide-type local anesthetic with a better safety profile compared with bupivacaine, lower cardiotoxicity, and less intense motor block.

This study aimed to evaluate patients undergoing laparoscopic sleeve gastrectomy who received OSTAP block with 0.2% ropivacaine compared with a control group with 0.9% saline solution, in terms of analgesic efficacy, opioid consumption, and inflammatory response.

## Methods

### Study design

This prospective, randomized, double-blind, clinical trial was conducted at a private hospital in São Luís, Maranhão, Brazil, between July 2025 and March 2026.

### Sample size

Using G*Power v. 3.1.9.4 (University of Düsseldorf) and a clinically significant difference of 3 points in pain intensity between groups on the verbal numerical rating scale, corresponding to a moderate effect size (d = 0.6), significance level (α) of 5%, statistical power (β) of 80%, and a 95% confidence interval (95%CI), the required sample size was calculated as 45 patients per group. An additional 10% was added to account for potential losses, yielding a total sample size of 100 patients.

### Randomization

The random allocation sequence was generated by computer using Randomizer®. Group assignments were placed in sealed envelopes that were opened on the day of surgery after participant enrollment and immediately before the assigned block. Participants were allocated 1:1 to the ropivacaine or saline group. An independent researcher who was not involved in patient recruitment or outcome assessment generated the random allocation sequence. A member of the research team screened and enrolled eligible participants. Immediately before the intervention, the anesthesiologist responsible for performing the block opened the sequentially numbered, sealed envelope and assigned the participant to the allocated intervention. Participants and postoperative outcome assessors were blinded to group assignment

### Inclusion and exclusion criteria

Patients aged 18–65 years old, American Society of Anesthesiologists (ASA) II-III, who were submitted for elective LSG were included. We excluded those with cardiac arrhythmias, cardiomyopathy, psychiatric disorders, liver disease, respiratory disease, neoplasia, cognitive impairment, preoperative use of analgesics, associated surgeries, hypersensitivity to local anesthetics, drug abuse, or perioperative blood transfusion.

### Clinical and anesthetic protocol

After anesthetic induction (fentanyl 5 mcg/kg, propofol 2–2.5 mg/kg, rocuronium 0.6 mg/kg) and orotracheal intubation, patients were managed according to the clinical protocol:

- Ropivacaine group (RG): bilateral OSTAP block with 0.2% ropivacaine (30 mL per side, total 60 mL);
- Control group (CG): bilateral OSTAP block with 0.9% saline solution, same volume.

A bilateral OSTAP block was performed using a convex transducer positioned in the subcostal region to visualize the external oblique, internal oblique, and transversus abdominis muscle planes, under ultrasound guidance, with injection in the plane between the transversus abdominis and internal oblique muscles^16^. Anesthesia was maintained with remifentanil and propofol titrated to maintain bispectral index (BIS) between 40 and 60.

### Data collection and instruments

Sociodemographic and clinical data were collected. Pain intensity at rest was assessed at 0, 1, 2, 4, 6, 12, and 24 h after extubation, whereas movement-evoked pain was assessed at 4, 6, 12, and 24 h, using a verbal numerical rating scale from 0 to 10. Time to first morphine request and the cumulative morphine dose consumed over 24 h were also recorded.

Postoperative nausea and vomiting (PONV) risk was assessed using the Apfel score^17^, which has been validated for the Brazilian population^18^. Quality of recovery was assessed using the Quality of Recovery-15 (QoR-15) questionnaire, validated for the Brazilian population^19^, applied on the first postoperative day.

Adverse effects of opioids and ropivacaine were recorded, including nausea, vomiting, dizziness, metallic taste, drowsiness, and perioral paresthesia. Hospital discharge criteria at the end of surgery were based on the protocol proposed by Mannaerts et al.^20^ and aligned with the enhanced recovery after bariatric surgery (ERABS) guidelines.

For interleukin (IL)-6 and IL-8 measurements, blood samples were collected in EDTA tubes from a peripheral vein in the upper limb contralateral to the surgical site before surgery (T1), 5 hours after the start of surgery (T2), and 24 hours after the end of surgery (T3). IL-6 and IL-8 concentrations were analyzed using enzyme linked immunosorbent assay (ELISA).

### Outcomes

The primary outcome was postoperative pain. Secondary outcomes included time to first morphine request, cumulative morphine consumption over 24 h, postoperative symptoms, QoR-15, functional recovery and discharge criteria, and IL-6 and IL-8 concentrations.

### Ethical aspects

The study was conducted in accordance with the Declaration of Helsinki and the Brazilian National Health Council Resolution No. 466/12. It was approved by the Research Ethics Committee of Hospital São Domingos (CAAE: 42177621.3.0000.5085; Opinion No. 4.821.253). Written informed consent was obtained from all participants. The trial was prospectively registered in the Brazilian Registry of Clinical Trials (ReBEC; RBR-6wz472d; UTN U1111-1277-1967) on July 19, 2025, and first patient enrollment on July 20, 2025, reported in accordance with the CONSORT 2025 statement.

### Statistical analysis

Data were analyzed using Statistical Package for the Social Sciences (version 21, Chicago, IL, United States of America). Normality was assessed with the Shapiro-Wilk’s test.

The prespecified analyses focused on between-group differences at each postoperative assessment time rather than on an overall longitudinal treatment effect. Therefore, the RG and CG were compared separately at each time point using the Mann–Whitney’s test for numerical outcomes and the χ^2^ or Fisher’s exact test for categorical outcomes. To account for multiplicity across repeated time-specific comparisons, *p*-values were adjusted using the Benjamini-Hochberg (BH) procedure within each outcome family. Both original and BH-adjusted p-values are reported, and statistical inference was based on BH-adjusted *p*-values, with values < 0.05 considered statistically significant.

Repeated-measures and mixed-effects models were not fitted. Consequently, the analyses did not model within-participant correlation or estimate overall time effects and group-by-time interactions.

## Results

In this study, 100 patients were screened and evaluated (Fig. 1).

**Figure 1:**
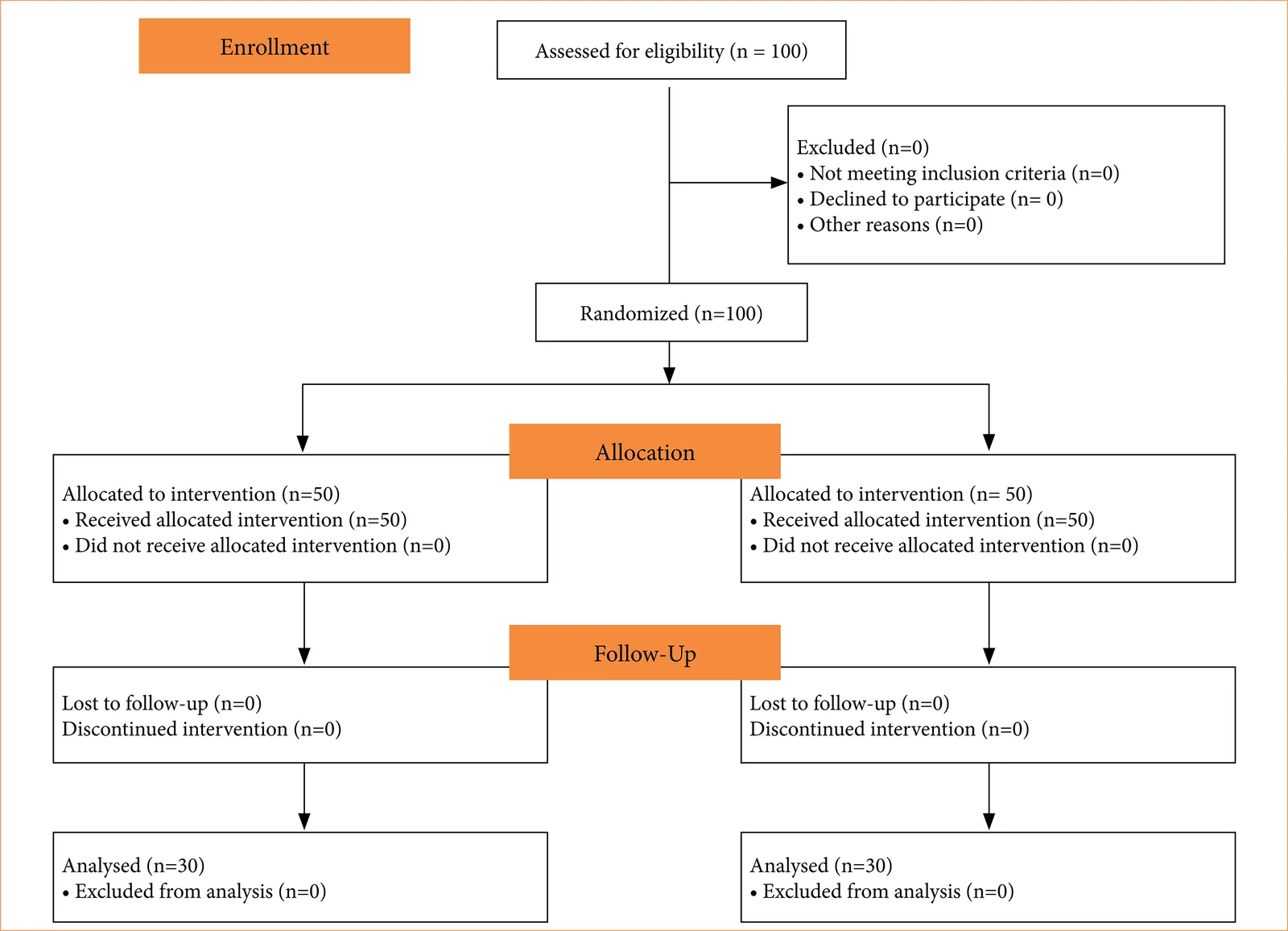
CONSORT flow diagram.

Source: Elaborated by the authors.

Women predominated in both groups (78%), with similar distributions regarding age, height, weight, body mass index, and ASA’s physical status classification. Most participants were classified as ASA II, corresponding to 96% in the RG and 98% in the CG. Median surgical duration was 77 min in the RG and 85 min in the CG, whereas median anesthesia duration was 137 and 131 min, respectively. No statistically significant between-group differences were identified in the evaluated baseline characteristics (Table 1).

**Table 1:** Demographic and clinical baseline data of patients undergoing sleeve gastrectomy.

Variables	RG	CG	*p* -value	BH adjusted *p* -value*
	Med (min–max)	Med (min–max)		
Age (years old)	33 (17–65)	36.5 (17–63)	0.082 §	0.459
**Gender, n (%)**				
Female	39 (78)	39 (78)	1.000 £	1.000
Male	11 (22)	11 (22)		
Height (cm)	165 (146–184)	166 (147–180)	0.614 §	0.702
Weight (kg)	100 (75–135)	96 (79–125)	0.225 §	0.459
Body mass index (kg/m^2^)	36.3 (31.2–46.1)	35.7 (31.0–42.2)	0.287 §	0.459
**ASA, n (%)**				
II	48 (96)	49 (98)	0.558 ¥	0.702
III	2 (4)	1 (2)		
Surgical duration (min)	77 (50–153)	85 (47–152)	0.174 §	0.459
Anesthesia duration (min)	137 (86–225)	131 (85–185)	0.235 §	0.459

ASA: American Society of Anesthesiology; RG: ropivacaine group; CG: control group;

§ Mann-Whitney’s test;

£ χ^2^ test;

¥ Fisher’s exact test;

* BH adjusted *p*-values were calculated using the Benjamini-Hochberg procedure within each predefined family of comparisons.

Source: Elaborated by the authors.

At rest, median pain scores were similar between groups at all evaluated time points, with no statistically significant differences. During movement (assessed at 4, 6, 12, and 24 h), the RG had a lower median pain score at 24 h, 1 (0–6) *versus* 2 (0–6) in the control group. However, the difference did not remain statistically significant after adjustment (*p* = 0.024; BH adjusted *p* = 0.096). Most patients (56%) requested morphine in the first postoperative hour, with similar distribution between groups. Total morphine consumption in 24 h was 10 mg (2–50) in the RG and 11 mg (1–30) in the CG, with no statistical difference (*p* = 0.926) (Table 2).

**Table 2:** Pain scores in patients undergoing laparoscopic sleeve gastrectomy with ropivacaine and without ropivacaine.

Variables	RG	CG	*p* -value	BH adjusted *p* -value*
	Med (min–max)	Med (min–max)		
**Postoperative pain at rest**				
0 h	5 (0–10)	6 (0–10)	0.876 §	0.992
1 h	5 (0–9)	5 (0–8)	0.992 §	0.992
2 h	4 (0–9)	4.5 (0–7)	0.538 §	0.942
4 h	4 (0–10)	4 (0–7)	0.411 §	0.942
6 h	2 (0–8)	3 (0–6)	0.238 §	0.833
12 h	2 (0–6)	2 (0–6)	0.137 §	0.833
24 h	5 (0–10)	6 (0–10)	0.876 §	0.992
**Postoperative pain in movement**				
4 h	4 (2–9)	5 (1–8)	0.604 §	0.635
6 h	4 (0–10)	4 (0–8)	0.635 §	0.635
12 h	2 (0–8)	3 (0–7)	0.129 §	0.258
24 h	1 (0–6)	2 (0–6)	0.024 §	0.096
**First morphine request, n (%)**				
Up to 1 h	29 (58)	27 (54)	0.527 £	0.926
Up to 2 h	20 (40)	23 (46)		
No morphine request	1 (2)	0 (0)		
Morphine consumption in 24 h (mg)	10 (2–50)	11 (1–30)	0.926 §	0.926

RG: ropivacaine group; CG: control group;

§ Mann-Whitney’s test;

£ χ^2^ test;

* BH adjusted *p*-values were calculated using the Benjamini-Hochberg procedure within each predefined family of comparisons.

Source: Elaborated by the authors.

Diet was resumed within 24 h in 98% of patients, and ambulation was early (< 12 h) in 76% of cases, with identical distribution between groups. Time to first flatus showed no significant differences. Regarding discharge criteria, 78% of patients had a visual analogue scale (VAS) < 4, 86% had no nausea or vomiting, 100% ambulated adequately, and 78% reported feeling confident about discharge, with no significant differences between RG and CG for any criterion (Table 3).

**Table 3:** Return of functions and discharge criteria in patients undergoing laparoscopic sleeve gastrectomy with and without ropivacaine.

Variables	RG	CG	*p* -value	BH adjusted *p* -value*
	n (%)	n (%)		
**Return to functions**				
**Diet**				
Up to 12 h	1 (2)	1 (2)	1.000 ¥	1.000
Up to 24 h	49 (98)	49 (98)		
**Flat passage (h)**				
Up to 12 h	22 (44)	18 (36)	0.683 £	1.000
Up to 24 h	16 (32)	17 (34)		
Up to 48 h	12 (24)	15 (30)		
**Ambulation (h)**				
< 12 h	38 (76)	38 (76)	1.000 £	1.000
12 h or more	12 (24)	12 (24)		
**Discharge criteria**				
**Medical history**				
VAS score < 4	41 (82)	37 (74)	0.334 £	0.501
No complaints of nausea or vomiting	44 (88)	42 (84)	0.564 £	0.564
Adequate ambulation	50 (100)	50 (100)	NA	NA
No complaints of calf pain	50 (100)	50 (100)	NA	NA
The patient feels confident about discharge	42 (84)	36 (72)	0.148 £	0.444
**Physical examination**				
No abdominal tension	50 (100)	50 (100)	NA	NA
Body temperature < 38°C	50 (100)	50 (100)	NA	NA
Heart rate < 100 bpm	50 (100)	50 (100)	NA	NA
SpO_2_ ≥ 95%	50 (100)	50 (100)	NA	NA

RG: ropivacaine group; CG: control group; VAS: visual analogue scale;

£ χ^2^ test;

¥ Fisher’s exact test;

NA: not applicable;

* BH adjusted *p*-values were calculated using the Benjamini-Hochberg procedure within each predefined family of comparisons.

Source: Elaborated by the authors.

Nausea was the most prevalent symptom, affecting 52% of the sample in the first hours and progressively decreasing to 9% at 24 h. Vomiting ranged from 11 (0 h) to 4% (24 h). Drowsiness was highly prevalent in the immediate postoperative period (81% at 0 h), with a gradual reduction to 23% at 24 h. Dizziness, metallic taste, and perioral paresthesia were reported at low frequencies (Table 4).

**Table 4:** Postoperative symptoms in patients undergoing laparoscopic sleeve gastrectomy with and without ropivacaine.

Variables	Group		*p* -value	BH adjusted *p* -value*
	RG	CG		
	n (%)	n (%)		
**Nausea**				
0 h	24 (48)	28 (56)	0.423 £	0.848
1 h	25 (50)	27 (54)	0.689 £	0.848
2 h	25 (50)	25 (50)	1.000 £	1.000
4 h	25 (50)	21 (42)	0.422 £	0.848
6 h	24 (48)	21 (42)	0.546 £	0.848
12 h	17 (34)	13 (26)	0.383 £	0.848
24 h	4 (8)	5 (10)	0.727 ¥	0.848
**Vomiting**				
0 h	7 (14)	4 (8)	0.338 £	0.372
1 h	8 (16)	3 (6)	0.110 £	0.372
2 h	7 (14)	4 (8)	0.338 £	0.372
4 h	5 (10)	1 (2)	0.092 ¥	0.372
6 h	10 (20)	6 (12)	0.275 £	0.372
12 h	5 (10)	8 (16)	0.372 £	0.372
24 h	1 (2)	3 (6)	0.307 ¥	0.372
**Dizziness**				
0 h	8 (16)	4 (8)	0.218 £	0.551
1 h	4 (8)	3 (6)	0.695 ¥	0.695
2 h	9 (18)	7 (14)	0.585 £	0.682
4 h	6 (12)	10 (20)	0.275 £	0.551
6 h	10 (20)	7 (14)	0.424 £	0.594
12 h	12 (24)	6 (12)	0.118 £	0.551
24 h	1 (2)	0 (0)	0.315 ¥	0.551
**Metallic taste**				
0 h	3 (6)	3 (6)	1.000 ¥	1.000
1 h	4 (8)	3 (6)	0.695 ¥	0.973
2 h	4 (8)	4 (8)	1.000 ¥	1.000
4 h	3 (6)	2 (4)	0.646 ¥	0.973
6 h	2 (4)	1 (2)	0.558 ¥	0.973
12 h	1 (2)	2 (4)	0.558 ¥	0.973
24 h	1 (2)	2 (4)	0.558 ¥	0.973
**Drowsiness**				
0 h	41 (82)	40 (80)	0.799 £	0.953
1 h	40 (80)	42 (84)	0.603 £	0.953
2 h	37 (74)	38 (76)	0.817 £	0.953
4 h	34 (68)	34 (68)	1.000 £	1.000
6 h	28 (56)	32 (64)	0.414 £	0.953
12 h	19 (38)	26 (52)	0.159 £	0.953
24 h	12 (24)	11 (22)	0.812 £	0.953
**Perioral paresthesia**				
0 h	0 (0)	1 (2)	0.315 ¥	0.315
1 h	0 (0)	0 (0)	NA	NA
2 h	0 (0)	0 (0)	NA	NA
4 h	1 (2)	0 (0)	0.315 ¥	0.315
6 h	1 (2)	0 (0)	0.315 ¥	0.315
12 h	1 (2)	0 (0)	0.315 ¥	0.315
c	0 (0)	1 (2)	NA	NA

RG: ropivacaine group; CG: control group; VAS: visual analogue scale;

£ χ^2^ test

¥ Fisher’s exact test;

NA: not applicable;

* BH adjusted *p*-values were calculated using the Benjamini-Hochberg procedure within each predefined family of comparisons.

Source: Elaborated by the authors.

Most patients (61%) had high PONV risk, and 11% had very high risk, with similar distribution between groups. The median QoR-15 score was identical between groups (118 points; *p* = 0.717), with 55% of patients classified as having good recovery quality. There were no statistically significant differences between groups for any evaluated category (Table 5).

**Table 5:** Assessment scales in patients undergoing laparoscopic sleeve gastrectomy with and without ropivacaine.

Variables	Groups		*p* -value	BH adjusted *p* -value*
	RG	CG		
	Med (min–max)	Med (min–max)		
**PONV**				
**Risk factors for PONV**				
Female sex	39 (78)	39 (78)	0.805 ¥	0.805
Non-smoker	46 (92)	45 (90)	0.727 ¥	0.805
History of PONV or motion sickness	50 (100)	48 (96)	0.153 ¥	0.612
Postoperative opioids	39 (78)	39 (78)	0.666 ¥	0.805
**Risk for PONV**				
Low	2 (4)	1 (2)	0.805 ¥	0.805
Moderate	10 (20)	15 (30)		
High	32 (64)	29 (58)		
Very high	6 (12)	5 (10)		
**QoR-15**				
Poor	1 (2)	4 (8)	0.514 ¥	0.514
Good	27 (54)	28 (56)		
Moderate	15 (30)	13 (26.		
Excellent	7 (14)	5 (10)		
QoR-152 total, med (min–max)	118 (89–146)	118 (86–146)	0.717 §	0.717

RG: ropivacaine group; CG: control group; PONV: postoperative nausea and vomiting; QoR-15: Quality of Recovery-15;

§ Mann-Whitney’s test;

£: χ^2^ test;

¥ Fisher’s exact test;

* Benjamini-Hochberg adjusted *p*-values were calculated using the Benjamini-Hochberg procedure within each predefined family of comparisons.

Source: Elaborated by the authors.

Both interleukins increased from T1 to T2 and decreased from T2 to T3. For IL-6, preoperative concentrations were numerically higher in the RG than in the CG (median 5.0 versus 3.5 pg/mL), but the difference was not statistically significant (*p* = 0.067; BH adjusted *p* = 0.201). For IL-8, T3 concentrations were numerically lower in the RG than in the CG (7.2 *versus* 9.8 pg/mL), but this difference was not statistically significant either (*p* = 0.073; BH adjusted *p* = 0.142) (Table 6).

**Table 6:** Interleukin levels in patients undergoing laparoscopic sleeve gastrectomy with and without ropivacaine.

Variables	Groups			Total	*p* -value §	BH adjusted *p* -value*
	RG	CG				
	Med (min–max)	Med (min–max)		Med (min–max)		
**Interleukin 6**						
T1 (pg/mL)	5.0 (0.0–13.1)	3.5 (0.0–14.1)		4.3 (0.0–14.1)	0.067	0.201
T2 (pg/mL)	10.6 (0.0.1–102.9)	13.8 (0.0–146.3)		11.8 (0.0–146.3)	0.929	0.929
T3 (pg/mL)	6.2 (0.0–29.3)	6.8 (0.0–60.7)		6.5 (0.0–60.7)	0.716	0.929
**Interleukin 8**						
T1 (pg/mL)	8.2 (0.1–41.6)	6.5 (0.0–88.5)		7.8 (0.0–88.5)	0.102	0.142
T2 (pg/mL)	10.1 (1.5–84.1)	8.8 (0.0–79.0)		9.4 (0.0–84.1)	0.142	0.142
T3 (pg/mL)	7.2 (0.6–27.1)	9.8 (1.3–18.6)		8.4 (0.6–27.1)	0.073	0.142

§ Mann-Whitney’s test;

* BH adjusted *p*-values were calculated using the Benjamini-Hochberg procedure within each predefined family of comparisons.

Source: Elaborated by the authors.

*

## Discussion

This randomized, double-blind, clinical trial evaluated whether adding 0.2% ropivacaine to bilateral OSTAP block improved analgesia, opioid consumption, recovery, and inflammatory response after laparoscopic sleeve gastrectomy. After adjustment for multiple comparisons, no statistically significant between-group differences were identified in pain scores, opioid consumption, functional recovery, postoperative symptoms, QoR-15, or cytokine concentrations. The lower movement-evoked pain score at 24 h observed in the unadjusted analysis did not remain statistically significant after adjustment and should not be interpreted as confirmatory evidence of analgesic efficacy.

### Sample profile

This study evaluated patients undergoing laparoscopic sleeve gastrectomy who received OSTAP block with 0.2% ropivacaine compared to a CG that received 0.9% saline solution.

The predominance of female sex, a body mass index of 35.8 kg/m^2^, and an ASA II classification are consistent with those reported in international bariatric surgery registries. The 9th IFSO Global Registry Report^21^ states that most patients undergoing bariatric-metabolic procedures worldwide are female, with a median body mass index of 36.1–47.65 kg/m^2^.

The median surgical duration of 80 min and anesthetic duration of 135 min are within the ranges reported in clinical trials. Shrief et al.^22^ reported a median surgical duration of 75–80 min in a randomized clinical trial of OSTAP in LSG. Zhou et al.^23^, evaluating opioid-free anesthesia with lateral TAP block in LSG, reported a surgical duration of 136.5 ± 31.2 min, which was higher than in the present study, possibly due to differences in institutional learning curves.

### Postoperative pain

Movement-evoked pain at 24 h was numerically lower in the RG in the unadjusted analysis (*p* = 0.024), but the difference was not statistically significant after Benjamini-Hochberg adjustment (BH adjusted *p* = 0.096).

The 0.2% ropivacaine concentration, which is lower than concentrations commonly evaluated in several TAP block trials, is one possible explanation for the absence of a detectable effect. Sun et al.^24^ in a meta-analysis of 19 randomized clinical trials including 1,217 patients showed that only 0.375 and 0.5% ropivacaine concentrations in TAP block were able to significantly reduce opioid consumption in the first 24 h postoperatively. In contrast, concentrations below 0.375% had limited efficacy in reducing opioid use. Zhang et al.^25^ reinforced this issue in a systematic review protocol on the optimal ropivacaine concentration for TAP block in cesarean section, reporting that concentrations from 0.25 to 0.75% are used in clinical practice, with possible superiority of higher concentrations in prolonging analgesia duration.

Toker et al.^26^, in a randomized controlled trial with 60 patients undergoing laparoscopic hysterectomy, demonstrated that bilateral OSTAP block with 0.375% bupivacaine (40 mL total) significantly reduced tramadol consumption in 24 h (77.33 ± 59.82 mg *versus* 125.00 ± 61.27 mg; *p* = 0.008) and reduced VAS scores at rest in the first 4 h after surgery, with significant differences at 0, 1, and 2 h. These findings suggest that local anesthetic concentration may influence the magnitude and duration of analgesia. However, differences in the anesthetic agent, surgical procedure, study design, and perioperative analgesic regimen preclude attributing the present results to concentration alone.

Huang et al.^27^, comparing OSTAP block with the transmuscular quadratus lumborum block (QLB) in laparoscopic hysterectomy, used 0.375% ropivacaine (20 mL per side) in both groups. The OSTAP group showed cumulative 24 h morphine consumption of 26.1 ± 13.3 mg, with incisional VAS scores significantly lower than preoperative values at all time points, but visceral pain scores were higher than in the QLB group. These findings suggest that OSTAP block, which primarily targets the somatic innervation of the abdominal wall, may provide less consistent control of visceral pain. This limited visceral coverage may partly explain the absence of between-group differences in pain at rest in the present study, although somatic and visceral pain components were not assessed separately.

Wu et al.^28^, in a pioneering study of 80 patients undergoing open radical gastrectomy, compared bilateral OSTAP block with 0.375% ropivacaine (20 mL per side) with thoracic epidural analgesia and intravenous opioids alone. In that study, pain scores with OSTAP were lower than with intravenous opioids, and morphine consumption was similar to that observed with epidural analgesia. These results suggest that OSTAP may provide effective analgesia in that specific surgical setting. However, differences in surgical approach, comparator groups, ropivacaine concentration, and perioperative care limit direct extrapolation to laparoscopic sleeve gastrectomy and to the 0.2% concentration used in the present trial.

Davey et al.^15^, in a meta-analysis specific to LSG with TAP block including 11 randomized clinical trials and 776 patients, confirmed significant reductions in pain scores up to 24 h, with a mean difference at 24 h of -0.77 (95%CI -1.42 to -0.12; *p* < 0.001), although the effect dissipated by 48 h. Although these findings provide context for the lower movement-evoked pain score observed at 24 h in the present study, its isolated occurrence and the absence of differences at earlier time points or in opioid consumption preclude confidently attributing it to a residual local anesthetic effect or to an interaction with multimodal analgesia.

### Opioid consumption

Groups showed similar total morphine consumption in 24 h. The literature generally reports lower consumption in patients receiving TAP block. Hamid et al.^14^, in a meta-analysis of 10 randomized clinical trials including 817 patients undergoing laparoscopic bariatric surgery, showed that TAP block with short-acting local anesthetics significantly reduced 24 h opioid consumption (standardized mean difference = -2.23 mg IV morphine equivalents; *p* < 0.001), with mean 24 h consumption of 29.24 mg (0.15–32.2 mg) among patients receiving TAP block, higher than in our study.

Grape et al.^13^, in a trial sequential meta-analysis including 13 studies and 1,025 bariatric patients, observed a significant but modest reduction in postoperative opioid consumption with TAP block.

In the study by Oliveira et al.^29^, with a smaller sample (n = 19) evaluating TAP block with ropivacaine on postoperative analgesia, no significant difference in opioid consumption was observed: the RG received 7.5 mg (interquartile range: 2.5–11.5) IV morphine equivalents *versus* 13 mg (7–21.5) in the saline group (*p* = 0.07). However, there was a trend toward lower use.

Davey et al.^15^ also found no significant difference in rescue analgesic consumption in LSG (*odds ratio* = 0.39; 95%CI 0.14–1.14; *p* = 0.090), consistent with the present study. The relatively low ropivacaine concentration is one possible explanation for the absence of an opioid-sparing effect, because concentrations below 0.375% showed less consistent effects in the meta-analysis by Sun et al.^24^. Rouholamin et al.^30^, in a double-blind trial with 200 women undergoing laparoscopic gynecologic surgery, demonstrated that TAP block with 0.5% ropivacaine (0.5 mg/kg) significantly reduced pain scores at all time points up to 48 h (*p* < 0.001) and decreased rescue meperidine use. With a concentration 2.5 times higher than in the present study, this reinforces the dose-response relationship of ropivacaine in TAP blocks. However, cross-study comparisons cannot establish a dose-response relationship. Differences in surgical procedures, analgesic regimens, block techniques, outcome definitions, and patient characteristics, as well as the possibility of no clinically relevant incremental effect, should also be considered.

In the present study, most patients requested morphine in the first postoperative hour, with similar distribution between groups. This high rate of early requests aligns with Abdelhamid et al.^31^, who compared the erector spinae plane block (ESPB) with the OSTAP block, both using ropivacaine, in obese patients undergoing LSG. In that study, the OSTAP group had a median time to first rescue analgesia of 75 (60–125) versus 165 min (90–300; *p* < 0.001) in the ESPB group, suggesting that blocks providing broader dermatomal coverage, including posterior dermatomes, may prolong time to first rescue analgesia.

### Functional recovery and discharge criteria

Functional recovery parameters, diet within 24 h, early ambulation (< 12 h), and time to first flatus were similar between groups, which may reflect the effectiveness of the ERABS protocol. Zhang et al.^32^ showed that TAP block significantly shortens time to first flatus (weighted mean difference = -5.17 h; 95%CI -8.58 to -1.77) and time to first ambulation (weighted mean difference = -0.46; 95%CI -0.92–0.00), but without impact on total length of hospital stay (standardized mean difference = -2.76 h; 95%CI -12.61–7.09). Davey et al.^15^ likewise found no difference in length of stay after LSG with TAP block (mean difference = -0.26 days; 95%CI -0.76–0.25; *p* = 0.320).

These data suggest that, in a structured ERABS setting, discharge criteria are more influenced by adherence to the institutional protocol than by regional analgesia type alone. Wong et al.^33^, using an ERABS protocol, also found no difference in length of stay when evaluating TAP block with liposomal bupivacaine in bariatric surgery (LSG and gastric bypass), reinforcing this finding.

### Nausea, vomiting, and adverse events

The high prevalence of nausea, drowsiness, and the distribution of high and very high PONV risk are characteristic of the bariatric population. Mieszczański et al.^34^, in a scoping review on PONV prevention in laparoscopic bariatric surgery, highlighted that PONV incidence in this population may reach 80%, substantially higher than in general surgery (30%), due to a combination of multiple Apfel risk factors (female sex, non-smoking status, prior PONV, and postoperative opioid use).

No statistically significant between-group differences in nausea or vomiting were identified in the present trial. This finding differs from Hamid et al.14, who reported lower PONV incidence with TAP block (*odds ratio* = 0.20; *p* = 0.01), and with Grape et al.^13^, who observed a significant reduction (relative risk = 0.57; 95%CI 0.33–0.98; *p* = 0.04). Similarly, Emile et al.^35^ reported significantly lower PONV scores with TAP block (2.1 ± 0.9 *versus* 3.0 ± 0.9; *p* < 0.001) in a randomized trial of laparoscopic bariatric surgery. Thus, regardless of the care protocol employed, both groups benefited from the block. Differences in the local anesthetic regimen, opioid exposure, antiemetic prophylaxis, outcome definitions, and baseline PONV risk may partly account for the discrepancy.

Symptoms potentially associated with local anesthetic exposure, including perioral paresthesia, metallic taste, and dizziness, were infrequent and did not differ significantly between groups, suggesting a drug’s safety profile at the dose and volume used. Babu and Mayilsamy^36^, comparing 0.375% ropivacaine and 0.25% bupivacaine for TAP blocks in laparoscopic abdominal surgery, found no block-related complications or systemic toxicity in either group, with similar VAS scores and analgesic consumption.

Vaddi et al.^37^ compared 0.5% ropivacaine with 0.25% bupivacaine for TAP blocks during abdominal surgery and concluded that ropivacaine provided slightly longer analgesia without significant adverse events. Similarly, Arunalatha et al.^38^, comparing 0.25% bupivacaine and 0.5% ropivacaine in TAP block for abdominal surgery under general anesthesia, showed that ropivacaine produced significantly longer analgesia (14.6 versus 10.3 h; *p* < 0.001) and lower rescue analgesic consumption, reinforcing the superiority of ropivacaine at adequate concentrations.

### Quality of recovery

Median QoR-15 scores classified recovery quality as good and within the expected ranges for intermediate-complexity surgeries, similar to the values reported by Stark et al.^39^, who in the original QoR-15 validation study found mean scores of 118 ± 20 for intermediate surgery and 92 ± 23 for major surgery. Additionally, Yang et al.^40^, comparing ESPB versus paravertebral block (PVB) in LSG, reported QoR-15 scores at 24 h of 119.0 ± 15.2 in the ESPB group *versus* 125.5 ± 14.2 in the PVB group (*p* = 0.014), suggesting that blocks with broader dermatomal coverage, including visceral components, may measurably improve recovery quality.

In Oliveira et al.^29^, there was a positive trend in global QoR-40 scores 24 h after surgery in the group receiving TAP block with ropivacaine, with median 175.5 (170–189) *versus* 170 (160–175) in the CG (*p* = 0.06).

The similarity in QoR-15 results across groups in the present study may reflect the instrument’s multidimensionality, which encompasses pain, emotional well-being, physical comfort, functional independence, and social support. Because no consistent between-group differences were identified across pain, opioid consumption, functional recovery, or postoperative symptoms after adjustment, the absence of a difference in the global QoR-15 score is consistent with the overall pattern of the trial. The unadjusted movement-pain difference at 24 h should not be treated as an established effect when interpreting QoR-15.

### Inflammatory markers

Interleukin kinetics showed the expected pattern of acute inflammatory response to surgical trauma, with an increase from T1 (preoperative) to T2 (5 h) and a decrease from T2 to T3 (24 h), with a trend toward lower IL-8 at T3 in the RG, but the difference was not statistically significant after Benjamini-Hochberg adjustment.

Liu et al.^41^, in a study of 65 patients undergoing radical gastrectomy for gastric cancer, showed that bilateral OSTAP block with 0.5% ropivacaine (20 mL per side) plus general anesthesia significantly reduced cortisol, norepinephrine, epinephrine, IL-6, and IL-10 levels at 4, 24, and 48 h postoperatively (*p* < 0.05 for all), confirming the ability of subcostal TAP block with ropivacaine to modulate neuroendocrine and inflammatory responses to surgical trauma. Notably, the ropivacaine concentration in that study (0.5%) was 2.5 times higher than in the present study (0.2%), which may explain the greater magnitude of the anti-inflammatory effect observed there.

Ropivacaine has documented *in-vitro* and *in-vivo* anti-inflammatory properties that extend beyond its local anesthetic effect^42-44^. In addition to blocking nerve conduction, amide-type local anesthetics, particularly ropivacaine, inhibit neutrophil migration and activation, reduce the release of proinflammatory mediators, and modulate intracellular signaling pathways involved in the inflammatory response^45,46^. These mechanisms may explain the trend toward lower IL-8 at T3 observed in this study, since IL-8 is a key chemokine in neutrophil recruitment to the site of inflammation.

Xu et al.^47^, in a review of opioid-free anesthesia within ERAS protocols for gastrointestinal surgery, emphasized that combining regional blocks with opioid-free strategies may enhance perioperative modulation of inflammation, reducing markers such as IL-6, tumor necrosis factor-α, and C-reactive protein. This reinforces the importance of including inflammatory biomarkers as outcomes in clinical trials of regional analgesia, as done in the present study.

### Limitations

This trial has limitations. It was conducted at a single center, which may limit generalizability. Although participants and postoperative outcome assessors were blinded, the anesthesiologist performing the block was aware of group assignment, which may have introduced performance bias. The analysis used separate between-group comparisons at each assessment time without repeated-measures or mixed-effects modeling; therefore, within-participant correlation, overall time effects, and group-by-time interactions were not estimated. The Benjamini-Hochberg procedure was applied within outcome families to address multiplicity, and no between-group comparison remained statistically significant after adjustment. The numerical baseline difference in IL-6 and the absence of a baseline-adjusted longitudinal cytokine analysis further warrant caution when interpreting the inflammatory outcomes.

## Conclusion

Among patients undergoing laparoscopic sleeve gastrectomy within a standardized multimodal analgesic pathway, adding 0.2% ropivacaine to bilateral OSTAP block was not associated with statistically significant between-group differences after Benjamini-Hochberg adjustment in pain scores, morphine consumption, postoperative symptoms, functional recovery, QoR-15, or IL-6 and IL-8 concentrations. The lower movement-evoked pain score at 24 h observed in the unadjusted analysis did not remain statistically significant and should not be interpreted as confirmatory evidence of efficacy. Adequately powered multicenter randomized trials with a prespecified primary outcome and assessment time, as well as longitudinal analyses that account for repeated measurements, are needed to determine whether specific ropivacaine concentrations provide clinically meaningful benefit.

## Data Availability

The data will be available upon request.
